# The Modern Role of Neonatal PICCs Subspecialty

**DOI:** 10.1111/nicc.70111

**Published:** 2025-07-03

**Authors:** Matheus Van Rens, Matthew Ostroff, Mohammad A. A. Bayoumi

**Affiliations:** ^1^ Neonatal Intensive Care Unit Radboud University Medical Center, Amalia Children's Hospital Nijmegen the Netherlands; ^2^ St. Joseph's Regional Medical Center Paterson New Jersey USA; ^3^ Neonatal Intensive Care Unit, Women's Wellness and Research Center, Hamad Medical Corporation Doha Qatar

**Keywords:** catheter safety, modified Seldinger technique, neonatal intensive care, neonatal PICC, simulation, VAMP framework, vascular access

## Abstract

This perspective review examines the evolving role of neonatal peripherally inserted central catheters (n‐PICCs) as a distinct subspecialty within neonatal vascular access. n‐PICCs are indispensable for delivering long‐term intravenous therapies in critically ill neonates. The review explores ethical considerations, equitable access to vascular access technologies and prioritisation of patient safety in procedural training. The implementation of specialised vascular access teams, rather than an all‐staff model, is advocated to enhance procedural success, reduce device‐related complications and promote a culture of accountability. Future directions include the development of smart catheter technologies, pseudo‐tunnelling techniques and simulation‐based education to advance and maintain clinical competency.

**Abbreviations**
CLABSI: central line‐associated bloodstream infections
CRBSI: catheter‐related bloodstream infections
MST: modified Seldinger technique
NICU: neonatal intensive care unit
n‐PICCs: neonatal peripherally inserted central catheters
PICCs: peripherally inserted central catheters
VADs: vascular access devices
VAMP: vascular access management plan

## 1 Brief Background to the Practice/Issue

Vascular access is a cornerstone of treatment in neonatal medical and surgical patients. It serves a wide range of critical purposes, including physiological monitoring, therapeutic interventions, supportive care, diagnostic imaging and procedural interventions for premature and ill neonates.

Neonatal Peripherally Inserted Central Catheters (n‐PICCs) represent a critical juncture in neonatal vascular access. While some may argue that n‐PICC placement is a straightforward technical procedure, others emphasise the skill and precision required, likening it to an artistic endeavour [1, 2]. This article highlights the practical advantages, technical considerations and challenges, as well as future directions of n‐PICC placement.

## 2 Brief Search Strategy and Review of the Evidence

A focused literature review was conducted to inform this perspective. PubMed, CINAHL and Web of Science were searched for English‐language articles published between January 2020 and March 2025 using the terms: ‘neonatal PICC’, ‘peripherally inserted central catheter’, ‘vascular access’, ‘neonatal intensive care’, ‘securement’, ‘simulation training’, and ‘Modified Seldinger Technique’.

Inclusion criteria were: (1) studies focused on neonatal populations, (2) evaluation of PICC‐related outcomes (e.g., infection, dislodgement, complications), (3) implementation of techniques (e.g., MST, ultrasound) and (4) discussion of team models or training.

Key high‐quality studies were selected based on methodological rigour, relevance to neonatal practice and recent publication (Table 1). These studies form the core evidence base supporting this review.

**TABLE 1:** Key evidence supporting best practices in neonatal PICC use.

Author (Year)	Study design	Population	Key intervention/focus	Findings
van Rens et al. (2022)	Retrospective cohort	NICU (*n* = 400)	Cyanoacrylate glue for PICC securement	Reduced dislodgement, no CRBSI
Barone et al. (2022)	Protocol development	Neonatal PICC insertions	Neo‐ECHOTIP + RAP protocol	Improved tip positioning and reduced malposition
Bayoumi et al. (2022)	Simulation programme report	Multicentre (Qatar)	5‐year simulation curriculum	Improved insertion success and team confidence
Ullman et al. (2020)	MAGIC consensus	Paediatric/neonatal	Appropriateness of PICC use	Recommended criteria for device selection
Sharpe et al. (2024)	Practice guideline	National (NANN)	Neonatal PICC care	Standardized dressing change, maintenance practices

### 2.1 Implementation Into Neonatal Intensive Care Unit (NICU) Nursing Practice

The integration of best evidence into NICU practice requires structured training, multidisciplinary collaboration and ongoing evaluation. Critical care nurses play a central role in implementing evidence‐based PICC practices by leading insertion protocols, maintaining catheter integrity and monitoring for complications. Establishing specialised vascular access teams led by experienced ICU nurses enhances procedural consistency and improves outcomes. Simulation‐based education should be embedded into unit‐level training to build technical and decision‐making skills, particularly in ultrasound‐guided insertion and the Modified Seldinger Technique. Unit protocols must reflect current standards, including aseptic insertion, securement with tissue adhesive and routine surveillance. Nurses should be empowered to participate in quality improvement initiatives and data collection to monitor success rates, dwell time and complication profiles. Institutional support for PICC‐specific certification and competency tracking is vital. By fostering a nurse‐led vascular access model, ICUs can improve safety, reduce central line‐associated infections and align practice with the evolving neonatal PICC evidence base.

### 2.2 Dressing Changes and Maintenance

Dressing changes are critical to n‐PICC maintenance [3, 4, 5]. Aseptic principles must continue during dressing changes, as outlined in the insertion protocol. Selecting appropriate dressings that provide secure adhesion, moisture control and breathability prevents infections and supports the overall stability of the catheter [1, 6].

The most commonly reported complications during maintenance include accidental dislodgement (20%–50%), phlebitis (2.8%–12%) and catheter‐related bloodstream infections (CRBSIs), which occur in 0.76%–4.7% of cases.

Occlusion is another major cause of catheter failure. n‐PICCs are typically 2 Fr or smaller and are used for continuous infusions rather than intermittent therapies. As such, they are not routinely locked. Instead, they are flushed after medication administration or other intermittent use. While consensus varies slightly between institutions, most protocols recommend routine flushing with 0.9% sodium chloride before and after medication administration and at set intervals (e.g., every 6–8 h if not in continuous use) [3, 6]. The use of heparinised saline remains debated; some centres avoid it due to risks of heparin‐induced thrombocytopaenia or drug incompatibility, whereas others may use low‐dose heparin (e.g., 10 units/mL) to prevent occlusion in select cases. Clinicians follow specific protocols for flushing, employing precise volumes and techniques to clear blood, medication residues and other obstructions from the catheter lumen. Adhering to these protocols with attention to detail ensures optimal catheter function and longevity [2, 7, 8, 9, 10].

### 2.3 Other Considerations

Beyond individual skills, the ‘art’ of n‐PICC placement is reinforced through standardised protocols, team‐based practice and continuous education. Standardised protocols, informed by evidence‐based guidelines, provide a framework for consistency while allowing for the adaptability needed in complex cases [4, 11]. Integrating specialised vascular access teams has been shown to improve patient outcomes by reducing catheter‐related complications and providing more consistent IV access care than general nursing staff [3, 6, 12]. Training programmes that combine theoretical knowledge, simulation exercises and bedside mentoring ensure that healthcare providers develop and maintain the high level of expertise required for these intricate procedures. Evidence supports the use of multimodal educational strategies in neonatal PICC training. These include structured curricula, hands‐on practice using mannequins or simulation models, supervised clinical placements and periodic competency assessments. Studies have shown that such approaches improve placement success rates, reduce complications and enhance team confidence. This multifaceted approach contributes to a consistent application of best practices, streamlined workflows and improved resource utilisation.

### 2.4 Challenges and Ethical Considerations

While n‐PICCs provide significant clinical and practical benefits, they also present unique challenges and ethical responsibilities. One of the foremost challenges is the accurate and safe placement of these catheters in fragile neonates, where repeated attempts can result in infiltration, vessel damage, arterial puncture or infection, thereby negating their intended therapeutic value [8, 13]. As discussed, real‐time ultrasound guidance is essential—not only for accuracy, but as an ethical imperative to minimise harm [4, 14]. Furthermore, high‐quality neonatal vascular access care extends beyond insertion to the timely recognition and management of complications such as extravasation and thrombosis. These may present subtly and be missed without structured assessment protocols. Neonatal nurses, advanced neonatal nurse practitioners (ANNPs) and neonatologists should be trained to detect early clinical signs, such as swelling, altered limb perfusion or localised discomfort, while leveraging point‐of‐care ultrasound (POCUS) to identify subclinical extravasation and confirm venous patency. In cases of suspected deep vein thrombosis (DVT), duplex Doppler ultrasound by certified neonatologists or radiologists is recommended. A multidisciplinary team approach involving vascular access specialists, radiology and neonatology ensures early intervention, minimises long‐term morbidity and upholds evidence‐based safety standards [1, 6, 15].

Ethical considerations centre around the balance between providing necessary treatment and minimising harm. The insertion of n‐PICCs is an invasive procedure, and informed consent from parents or caregivers is critical. Parents must receive comprehensive information about the potential risks, including strategies to mitigate them, along with the benefits and available alternatives to support informed decision‐making. Transparent communication fosters trust and reinforces the principles of family‐integrated care [9, 16].

Another ethical challenge is equitable access to n‐PICC technology and expertise. Resource‐limited settings may face barriers such as insufficient infrastructure, a shortage of trained personnel or a lack of necessary equipment, limiting the effective implementation of n‐PICC programmes. However, advancements in digital communication, including internet‐based consultations, video conferences and remote training, have facilitated the global dissemination of education and skill development, helping to bridge these gaps in practice. This disparity raises questions about fairness in neonatal care and highlights the need for global efforts to standardise access to advanced vascular access solutions [13, 17].

Finally, the ethical obligation to prevent complications, such as CLABSI, underscores the importance of adhering to evidence‐based practices. While achieving zero CLABSI rates is possible, it requires rigorous adherence to insertion and maintenance protocols, continuous monitoring and quality improvement initiatives. Premature privileging of trainees, fellows and vascular access team members to insert central lines should be avoided due to patient safety concerns, which should always be a priority over any training purposes. New trainees, fellows and vascular access team members should follow a strict pathway to get released to insert neonatal PICCs independently without impacting patient safety at any stage. This privileging process should follow the six steps of the pedagogical pathway for procedural skills training. This starts with the learn, see, practice, prove, do and maintain approach for the PICC insertion, as well as any other procedural skill training in neonatology [12, 18, 19, 20, 21]. All healthcare providers must remain vigilant and committed to minimising risks while delivering optimal care.

By addressing these practical and ethical challenges, clinicians can ensure that the implementation of n‐PICCs aligns with the highest standards of neonatal care. This dual focus on technical excellence and ethical responsibility fosters trust, improves outcomes and supports the broader goal of advancing neonatal healthcare globally.

## 3 Implementation

Implementation and future directions should prioritise advancing research, incorporating innovative technologies and methodologies and refining clinical practices to continuously improve patient outcomes.

- Education and Research: Further research and simulation‐based training should focus on the development and validation of assessment tools to evaluate PICC insertion proficiency across cognitive (knowledge), psychomotor (technical) and behavioural (communication) domains. Additionally, efforts should be made to bridge the gap between simulated PICC insertion performance and real‐world clinical outcomes in the NICU. Beyond technical skill acquisition, future simulation‐based curricula and research should explore the role of simulation‐based education in advancing vascular access team leadership, optimising human and system performance and improving risk and resource management in neonatal vascular access. More focus on timing‐focused vascular access research is a topic for future research and quality improvement projects.
- Specialised Vascular Access Teams: Establishing and expanding the implementation of specialised, trained vascular access teams within the NICU for PICC insertion is essential for improving patient safety and procedural outcomes.
- Smart catheter systems: Developing catheters with embedded sensors to monitor position, flow and potential complications in real‐time can revolutionise neonatal vascular access.
- Data‐driven quality improvement: Utilising real‐time data monitoring systems and registries to track outcomes, set key performance indicators and identify areas for continuous improvement can enhance care delivery.

## Author Contributions

M.V.R. did the literature search and wrote the first draft of the manuscript. M.O. and M.A.A.B. contributed to the literature search, co‐wrote the manuscript and reviewed it for intellectual and clinical input. All authors agreed on the submitted version.

## Ethics Statement

The authors have nothing to report.

## Consent

The authors have nothing to report.

## Conflicts of Interest

The authors declare no conflicts of interest.

## Data Availability

Data sharing not applicable to this article as no datasets were generated or analysed during the current study.
